# Fostering Freedom and Autonomy in Everyday Life in Green Care Farms

**DOI:** 10.1093/geroni/igaf122.1152

**Published:** 2025-12-31

**Authors:** Gijs Steinmann, Hilde Verbeek, Maud Hamers, Bram de Boer

**Affiliations:** Maastricht University, Maastricht, Limburg, Netherlands; Maastricht University, Maastricht, Limburg, Netherlands; Maastricht University, Maastricht, Limburg, Netherlands; Maastricht University, Maastricht, Limburg, Netherlands

## Abstract

Green Care Farms (GCFs) integrate physical, social, and organizational aspects to promote autonomy and active daily living for people living with dementia, combining care with agricultural activities. While environmental designs have long been recognized as crucial for promoting autonomy and well-being, research has largely overlooked the organizational dimension of care. This study examined how specific organizational aspects of GCFs foster freedom and autonomy in the everyday lives of people with dementia. Ethnographic fieldwork at a Dutch GCF was conducted, including participant observations and informal conversations. Observational data was complemented with semi-structured interviews, focusing on staff and their reasoning behind work-related actions and decisions. The coding process involved open coding of observation reports and interviews, thematic coding to develop a codebook, axial coding informed by organizational theory, and abductive selective coding to refine our interpretation. Results found that the GCF environment triggered the expression of autonomy in three ways: by stimulating physical activity to retain physical functioning; by encouraging participation in meaningful activities; and by enabling residents to enjoy freedoms and risks. Findings indicated a critical role of organizational aspects in enabling autonomy and freedom. Staff mindsets and behavioral norms acted as environmental presses, fostering physical activity and purposeful engagement. Moreover, shared norms and values were central to promote freedom. Other care settings may transfer these principles to their context. Overall, the GCF’s organizational culture was characterized by a distinct belief among staff that they did not work in a nursing home—implying that residents did not live in one either.

